# Endoscopic ultrasound-guided choledochoduodenojejunostomy via afferent limb: a novel approach for hepaticojejunostomy stricture

**DOI:** 10.1055/a-2748-1420

**Published:** 2025-12-08

**Authors:** Nozomi Okuno, Kazuo Hara, Shin Haba, Takamichi Kuwahara, Shimpei Matsumoto, Hiroki Koda, Tomoki Ogata

**Affiliations:** 1 538357 Department of Gastroenterology, Aichi Cancer Center, Nagoya, Japan

The efficacy of balloon-assisted endoscopic therapy for the hepaticojejunostomy anastomotic stricture (HJAS) has been reported 1 ; however, the procedure can be technically challenging. EUS-guided hepaticogastrostomy (EUS-HGS) has been described as an antegrade approach to the HJAS 2 3 , but when traversal is unsuccessful, lifelong stent exchanges may be required.

We report a case of the HJAS successfully managed by creating a new anastomosis through EUS-guided choledochoduodenojejunostomy (EUS-CDJS) from the duodenal bulb ( Video 1 ). A man in his fifties underwent cholecystectomy, extrahepatic bile duct resection, and biliary reconstruction for gallbladder cancer. He remained recurrence-free for 18 months; however, he was admitted with acute cholangitis due to the HJAS ( Fig. 1 ). EUS-HGS achieved initial management. After improvement, we planned antegrade treatment through the HGS route; however, cholangioscopy revealed that the anastomosis was completely scarred, precluding traversal ( Fig. 2 ).

A new anastomosis was created using EUS-CDJS for the hepaticojejunostomy anastomotic stricture that could not be traversed from the HGS route. EUS-CDJS, EUS-guided choledochoduodenojejunostomy; HGS, hepaticogastrostomy.Video 1

**Fig. 1:**
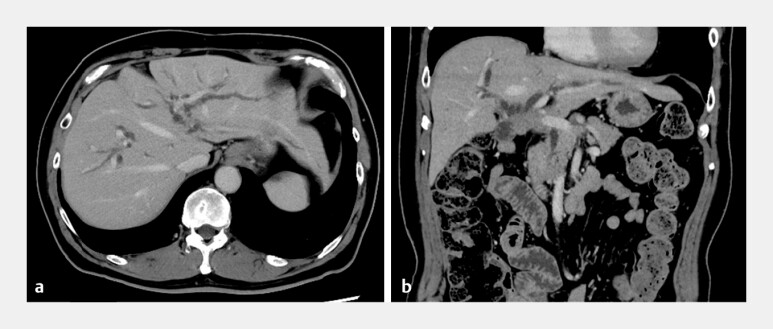
**a** A computed tomographic (CT) image at the time of admission for acute cholangitis shows bilateral intrahepatic bile duct dilatation. **b** A coronal CT image shows bilateral intrahepatic bile duct dilatation due to the hepaticojejunostomy anastomotic stricture.

**Fig. 2:**
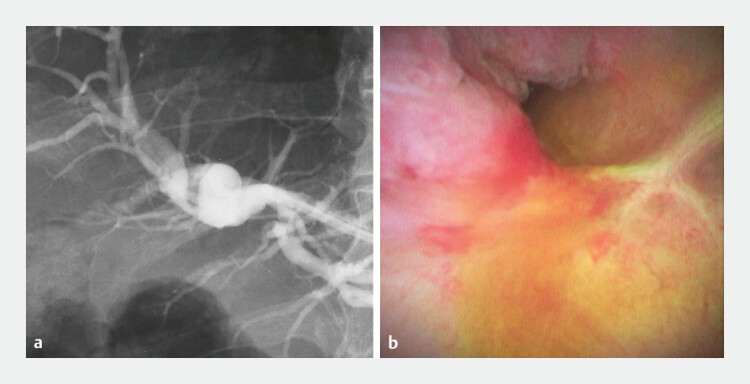
**a** Cholangiography via the HGS route shows no contrast flow from the anastomosis into the afferent limb. **b** Cholangioscopy via the HGS route shows complete scarring at the anastomosis, hindering guidewire passage. HGS, hepaticogastrostomy.

We therefore performed EUS-CDJS to create a new anastomosis ( Fig. 3 ). The HJAS was punctured from the duodenal bulb toward the posterior duct with a 19-gauge FNA needle via the afferent limb. The tract was dilated with a drill dilator (Tornus25; Asahi Intecc, Japan), and a fully covered self-expandable metal stent (HANAROSTENT Benefit 6 mm 10 cm; Boston Scientific, USA) was placed. No adverse events occurred, oral intake was resumed next day, and the patient was discharged on postoperative day 4.

**Fig. 3:**
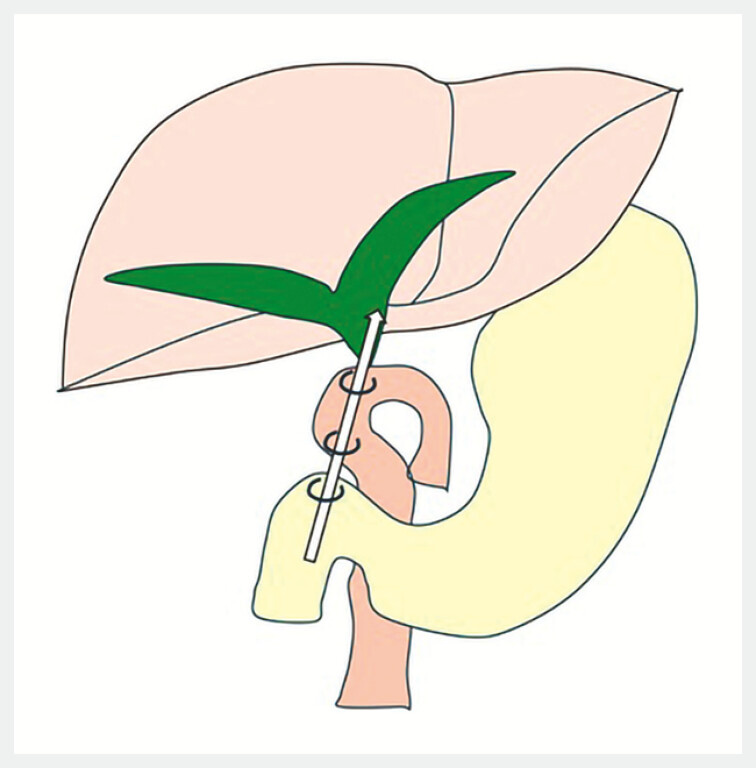
Schematic illustration of EUS-guided choledochoduodenojejunostomy (EUS-CDJS). Only a puncture route via the afferent limb is available, and the hepaticojejunostomy anastomosis is punctured from the duodenal bulb through the afferent limb to create a new anastomosis.

Two months later, the stent was upsized to 8 mm (HANAROSTENT Biliary Multi-hole) to dilate the anastomosis, and a plastic stent was placed in the contralateral duct to avoid unilateral obstruction. Three months thereafter, all stents were removed, and the new anastomosis had matured. Biliary scintigraphy confirmed adequate drainage ( Fig. 4 ).

**Fig. 4:**
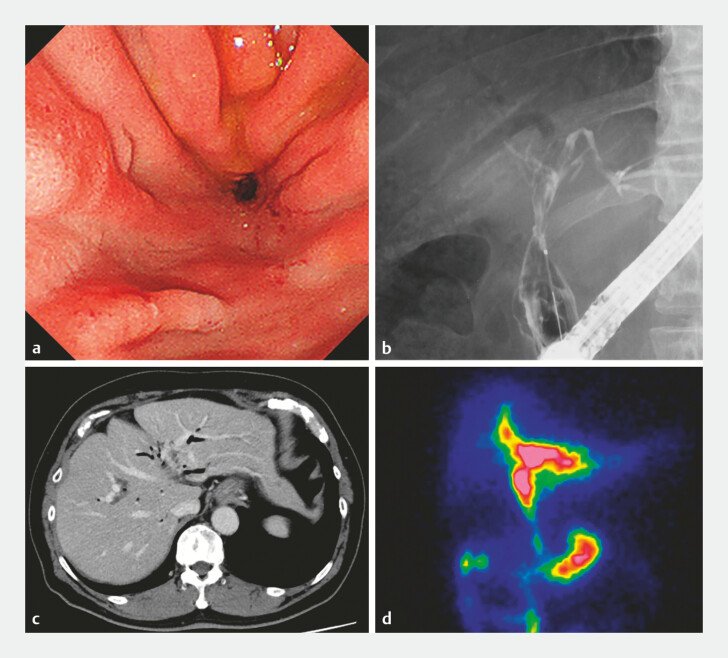
**a** An endoscopic image after complete removal of all stents shows the newly matured choledochoduodenojejunal anastomosis. **b** Cholangiography performed through the new anastomosis demonstrates satisfactory bile drainage. **c** Computed tomography demonstrates reduced dilatation of the intrahepatic bile ducts and the presence of pneumobilia. **d** Biliary scintigraphy demonstrates satisfactory bile outflow through the new anastomosis.

Although the usefulness of EUS-CDJS for benign distal biliary strictures has been reported 4 5 , to our knowledge, this is the first report describing the creation of a new anastomosis via the EUS-CDJS route through the afferent limb for the HJAS.

Endoscopy_UCTN_Code_TTT_1AS_2AH

## Notes

Endoscopy E-Videos https://eref.thieme.de/e-videos
*E-Videos* is an open access online section of the journal *Endoscopy* , reporting on interesting cases and new techniques in gastroenterological endoscopy. All papers include a high-quality video and are published with a Creative Commons CC-BY license. Endoscopy E-Videos qualify for HINARI discounts and waivers and eligibility is automatically checked during the submission process. We grant 100% waivers to articles whose corresponding authors are based in Group A countries and 50% waivers to those who are based in Group B countries as classified by Research4Life (see: https://www.research4life.org/access/eligibility/ ). This section has its own submission website at https://mc.manuscriptcentral.com/e-videos .
